# Electronic health record note review in an outpatient specialty clinic: who is looking?

**DOI:** 10.1093/jamiaopen/ooab044

**Published:** 2021-07-31

**Authors:** Jimmy S Chen, Michelle R Hribar, Isaac H Goldstein, Adam Rule, Wei-Chun Lin, Haley Dusek, Michael F Chiang

**Affiliations:** 1 Department of Ophthalmology, Casey Eye Institute, Oregon Health & Science University, Portland, Oregon, USA; 2 Department of Medical Informatics and Clinical Epidemiology, Oregon Health & Science University, Portland, Oregon, USA; 3 National Eye Institute, National Institutes of Health, Bethesda, Maryland, USA

**Keywords:** electronic health records, medical record systems—computerized, ambulatory care, outpatient clinics, hospital

## Abstract

Note entry and review in electronic health records (EHRs) are time-consuming. While some clinics have adopted team-based models of note entry, how these models have impacted note review is unknown in outpatient specialty clinics such as ophthalmology. We hypothesized that ophthalmologists and ancillary staff review very few notes. Using audit log data from 9775 follow-up office visits in an academic ophthalmology clinic, we found ophthalmologists reviewed a median of 1 note per visit (2.6 ± 5.3% of available notes), while ancillary staff reviewed a median of 2 notes per visit (4.1 ± 6.2% of available notes). While prior ophthalmic office visit notes were the most frequently reviewed note type, ophthalmologists and staff reviewed no such notes in 51% and 31% of visits, respectively. These results highlight the collaborative nature of note review and raise concerns about how cumbersome EHR designs affect efficient note review and the utility of prior notes in ophthalmic clinical care.

## INTRODUCTION

Electronichealth records (EHRs) have become an integral part of healthcare over the past decade. Between 2008 and 2017, EHR adoption more than doubled, with 85.9% of all outpatient physicians in the United States using an EHR as of 2017.^1^^,^^2^ While physicians acknowledge many benefits to EHR use,^3–5^ they also find EHRs time-consuming and difficult to use,^6–8^ hindering productivity,^9–11^ and contributing to provider burnout.^12–15^

Providers spend much of their time writing and reviewing documentation in EHRs, especially clinical notes.^16–18^ To minimalize time spent writing notes, many providers use content-importing technologies (ie, copy–paste, templates) to generate large chunks of text,^19^^,^^20^ but this practice can produce longer^21^ and more redundant notes.^22–25^ Some clinics have also implemented team-based documentation, involving ancillary staff in note entry. Providers report increased clinical efficiency and quality of care in both primary care^26–28^ and specialty clinics^21^^,^^29^ when practicing team-based documentation. However, studies have shown that scribes, one type of ancillary staff,^30^^,^^31^ document with significant variability.^32^ Less is known about how providers cope with the time demands of reviewing clinical notes or involve ancillary staff in this process. While previous work in inpatient settings has shown physicians preferentially review notes’ assessment and plan sections and primarily review notes written in the last 24 h,^33^^,^^34^ little is known about note review practices in outpatient settings, particularly in specialty care.

The purpose of this study was to address this knowledge gap by examining the EHR note review practices of physicians and ancillary staff in a specialty setting in ophthalmology. We hypothesized that both ophthalmologists and staff perform minimal manual note review. To test this hypothesis, we conducted a case study of note review practices in an academic outpatient ophthalmology clinic. This study helps illuminate ophthalmologists and ancillary staff note review practices that may have broader implications in ophthalmology and other specialties for EHR design, quality of care, and policymaking.

## METHODS

### Study setting

Oregon Health & Science University (OHSU) is a large academic medical center in Portland, Oregon with over 50 faculty ophthalmologists who together perform over 130 000 outpatient eye exams annually. In 2006, OHSU implemented an institution-wide EHR (EpicCare; Epic Systems) for all practice management, documentation, order entry, and billing. This study was approved by the institutional review board at OHSU and adheres to the Declaration of Helsinki.

### Dataset

We extracted data from OHSU’s clinical data warehouse for office visits completed between January 1, 2015, and December 31, 2017, in 9 ophthalmology subspecialties (Table 1). For each visit, we obtained visit information (type of visit, time of the visit, check-in and check-out time, diagnosis), a list of all prior notes available for the patient (including note type, date, and department), and audit log entries starting 3 days before the visit and ending 3 days after the visit was closed in the EHR. Audit log entries are data generated by user interactions with the EHR that include identifiers of prior notes reviewed before, during, and after visits. We included both 3 days prior to and after the visit was closed to capture as many relevant chart review activities as possible. Visits were included if the visit: (1) was a follow-up visit rather than a new patient or post-op visit, (2) was the patient’s most recent office visit, (3) was for 1 of the 3 most common visit diagnoses for that subspecialty, and (4) had complete data.

**Table 1.** Dataset characteristics

	Totals during study period			Included in study
Subspecialty	Physicians	Office visits	Patients	Office visits^a^
Comprehensive	3	29 253	10 426	2081
Pediatrics	3	21 470	7924	2193
Cornea	3	26 127	6662	1095
Retina	2	22 189	4474	1541
Neuro	2	8927	4887	459
Oculoplastics	2	11 966	4948	603
Uveitis	2	4416	1173	352
Glaucoma	2	12 988	2021	1180
Genetics	2	2430	1145	271
Overall	21	139 766	43 660	9775

*Note*: Overall, 9775 visits for 9775 patients from 21 ophthalmologists across 9 specialties in 2015–2017 were included in our study. Office visits were included if they were the most recent follow-up visit for each patient who had 1 of the 3 most common diagnoses in each subspecialty. All other notes for each patient were analyzed by whether they were reviewed during the included office visit.

^a^ Only the most recent office visit was included for each patient in our study.

### Note analysis

All data processing and statistical analysis were performed using R (version 3.6.0).^35^ For each included office visit, we identified all unique prior notes reviewed during that visit. Each audit log entry for a reviewed note identified the user ID, the note ID, and note access time. For each reviewed note, we identified (1) note type, (2) department, (3) user role, and (4) chronological visit order among prior office visits. The *note type* was either a prior office visit note or non-office note (procedures, photography, telephone notes, etc.). The *department* of the note was the clinical specialty for which the note was created, labeled as either ophthalmology or not. The *user role* was defined as attending physician, ancillary staff (technicians), or trainee (residents or fellows). The *visit order* was defined as the rank of prior office visits’ notes and was calculated only for ophthalmology office visit notes. Notes from the most recent prior visit were given a visit order of 1; notes from the visit 2 visits ago were given a visit order of 2, and so on.

Grouping note accesses by note type and department, we calculated the average number and percentage of notes accessed per visit relative to the total number of notes available for the patient. Notes accessed for each visit were further stratified as accessed by ancillary staff only, physician only, or both. Differences in number of notes reviewed by note types and user roles were analyzed using independent 2-group Mann–Whitney *U* tests and Pearson’s χ^2^ tests, with significance defined as *P *<* *.05. Bonferroni corrections were performed to correct for multiple comparisons.

## RESULTS

### Notes dataset

There was a total of 139 766 office visits for 43 660 patients during the study period, of which 9775 unique office visits for 9775 patients met inclusion criteria (Table 1). We excluded patients and visits that did not meet inclusion criteria to ensure analysis of “typical” ophthalmology follow-up visits. Overall, 21 ophthalmologists from 9 ophthalmology subspecialties were represented. The number of office visits included across each subspecialty ranged from 271 to 2193 visits.

### Notes reviewed by user role, note type, and department

Table 2 summarizes notes accessed per visit by user role, note type, and department. Results are shown for all notes and ophthalmology-specific office visit notes reviewed. Ancillary staff and trainees accessed significantly more notes per visit compared to ophthalmologists (2.1 ± 3.3 vs 1.3 ± 2.8, *P* < .001 for staff and 1.5 ± 3.3 vs 1.3 ± 2.8, *P* < .001 for trainees). For all user roles, the majority of office visit notes reviewed were ophthalmology notes rather than those written in other specialties. Ophthalmologists reviewed a median of zero ophthalmology office visit notes per visit, while ancillary staff and trainees reviewed a median of 1.

**Table 2.** Mean and median of notes reviewed by role and note type

Role	Notes reviewed					
	All notes			Ophthalmology office visit notes		
	Mean ± SD	Median	Mean ± SD (%)	Mean ± SD	Median	Mean ± SD (%)
Ophthalmologist	1.3 ± 2.8	1	2.6 ± 5.3	0.8 ± 1.4	0	8.3 ± 14.9
Ancillary staff	2.1 ± 3.3^a^	2	4.1 ± 6.2	1.1 ± 1.2^a^	1	10.1 ± 14.6
Trainees	1.5 ± 3.3^a^	1	2.5 ± 6.1	1.0 ± 1.9^a^	1	7.4 ± 13.9

*Note*: The mean number and median of notes reviewed per office visit were analyzed by roles defined as physician, ancillary staff, and trainees, as well as the type of note reviewed. Ophthalmology office visit notes included all prior office visit notes written by an ophthalmology physician.

*Abbreviation*: SD: standard deviation.

a Number of notes reviewed was significantly different compared to the number of notes reviewed by physicians (*P* < .001, 2-group Mann–Whitney *U* test with Bonferroni correction).

**Figure 1.**
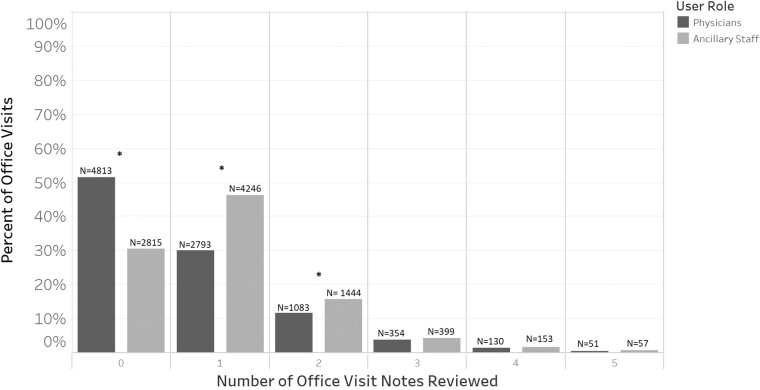
Histogram of the number of notes reviewed. The *x*-axis is the total number of prior office visit notes reviewed and the *y*-axis is the percent of all office visits that reviewed that number. Office visits with greater than 5 notes reviewed were not included in this figure. Starred bars (*) represent significantly different numbers of office visit notes reviewed between physicians and ancillary staff (*P* < .001, Pearson’s χ^2^ test with Bonferroni correction).

As shown in Figure 1, ophthalmologists reviewed zero ophthalmology office visit notes in 51.6% of office visits and 1 note in 30.2% of the office visits, while ancillary staff reviewed zero ophthalmology office visit notes in 30.5% of the visits and 1 note in 46.4% of the visits. Both ophthalmologists and ancillary staff infrequently reviewed more than 1 office visit note per office visit. Trainees were excluded from this analysis because they were not present for all included office visits.

### Notes reviewed by visit order

**Figure 2.**
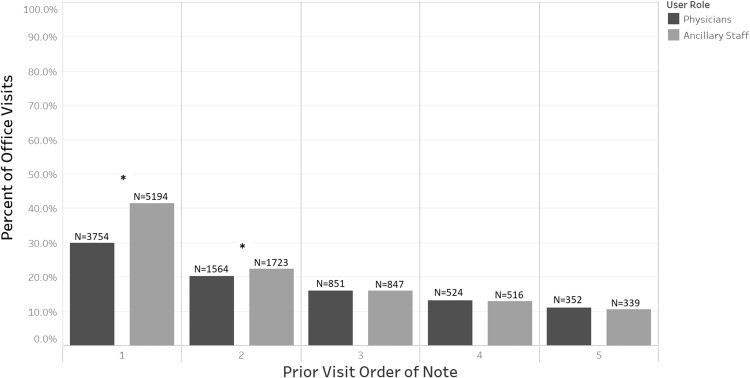
Percentage of notes reviewed from each prior visit. Percentages of office visits (*y*-axis) in which an ophthalmology office visit note was reviewed for each prior visit (*x*-axis). Each prior visit has a visit order defined as the number prior to the current visit (ie, visit order 1 = most recent prior office visit, 2 = second most recent, etc.). Each percentage represents the proportion of visit notes of that order that were reviewed (labeled above each bar) out of the total number of available notes of that visit order. Visits that were older than the fifth prior visit not included in the figure. Starred visit orders (*) represent proportions of ophthalmology office visit notes reviewed that were significantly different between physicians and ancillary staff (*P* < .001, Pearson’s χ^2^ test with Bonferroni correction).

Sorting prior ophthalmology office visit notes by visit order, Figure 2 shows that the most recent note (visit order = 1) was reviewed in 29.9% of office visits by ophthalmologists and 41.4% of visits by ancillary staff. Only the number of the most recent and second most recent office visit notes reviewed differed significantly between ancillary staff and ophthalmologists (*P* < .001 for both comparisons, Pearson’s χ^2^ test with Bonferroni correction). Notes with a visit order ≥3 were reviewed in less than 20% of visits by either ophthalmologists or ancillary staff.

## DISCUSSION

This study, which expands on an initial smaller-scale analysis,^36^ has 2 key findings: (1) note review was minimal by both ophthalmologists and ancillary staff and (2) note review is a collaborative activity with ancillary staff reviewing more notes than ophthalmologists.

**Figure 3.**
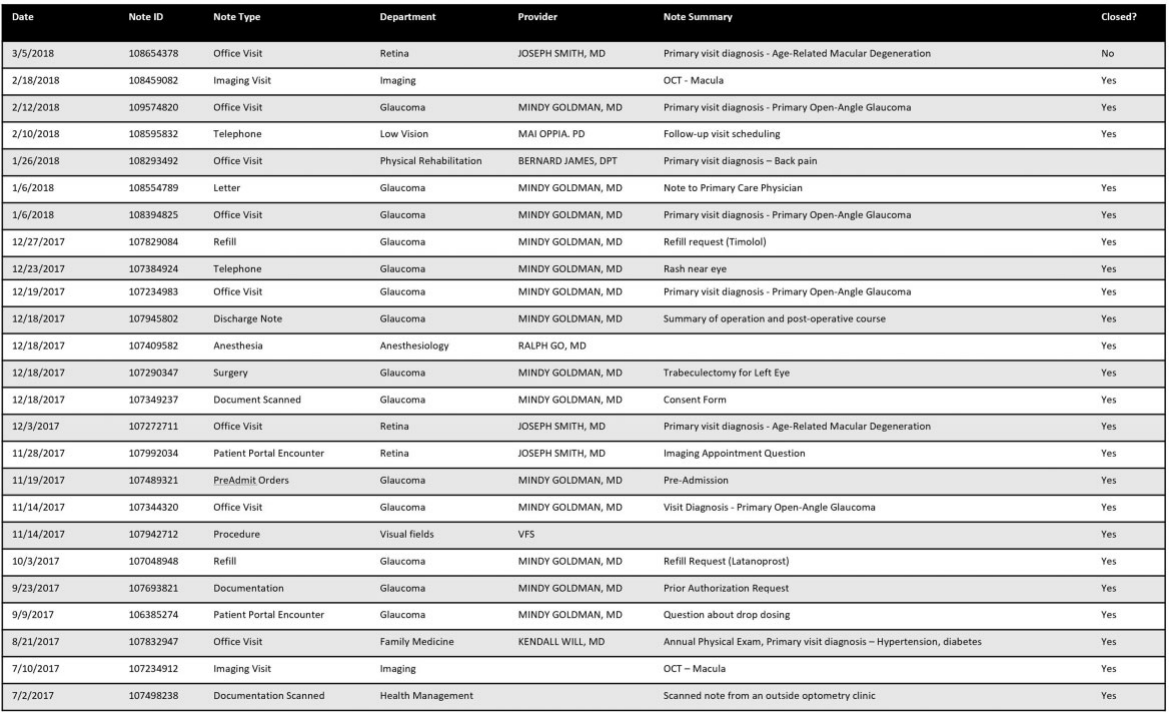
Sample note review interface. An example note review interface containing de-identified data filtered for notes specific to the ophthalmology department is shown. Of the notes available in this current view, 8 notes (32%) are office visit notes and 17 notes are non-office visit notes such as telephone, imaging, and surgery notes. Despite filtering for notes specific to ophthalmology, patients with more frequent visits and longer medical histories will often have long lists of notes available for review.

The first key finding is that note review is minimal by both ophthalmologists and ancillary staff. In 51.6% and 30.2% of office visits, ophthalmologists and ancillary staff did not review any prior ophthalmology office visit notes (Figure 1). When notes were reviewed, both roles almost exclusively reviewed the most recent ophthalmology visit note, corroborating work in the inpatient setting.^33^ These findings do not imply inadequate note review or clinical care by physicians or staff, instead, they are a result of EHR usage patterns that merit further studies. It is possible that current note review interfaces are not preferred sources due to the overload of fragmented information across multiple sources of data,^37^ which hinder efficient note review during visits. For example, Figure 3 shows a generic note review interface where office note visits are shown with similar importance to other note types, which is time-consuming to navigate even when filtered specifically for ophthalmology visits. To provide effective clinical care and mitigate inefficiencies associated with these interfaces, providers often streamline their note review by using note templates that aggregate structured patient information from the chart rather than manually reviewing prior notes, although this can result in longer notes that are more time-consuming to subsequently manually review. While user-centered EHR designs^38–41^ (ie, commercial, ophthalmology-specific EHRs) have been created to display patient data over time, further research is needed to understand the completeness of chart review using these tools. An important limitation of our study is the generalizability of our findings. Our study evaluated note review patterns in an integrated EHR at a single academic clinic and represents the first step in quantifying note review practices in outpatient care. Since, ophthalmologists often see patients for a small set of chronic problems, as opposed to multiple acute and chronic problems in primary care clinics, note review practices may vary. Future studies examining note review practices in other EHR systems, institutions, and specialties are needed.

The second key finding is that note review is collaborative, with ancillary staff reviewing more notes than ophthalmologists and having a significant role in chart review. Further analysis of all unique notes reviewed showed that 20.1% of all reviewed notes were reviewed by only the ophthalmologist, while 38.9% of notes were reviewed by only ancillary staff (data not shown). At our ophthalmology clinics, ancillary staff generally initiate an office visit note after their own chart review, which physicians then edit. If ancillary staff in a team-based model are reviewing more unique information than physicians, inclusion and exclusion choices by staff likely impact physician decision-making. Prior literature is missing on chart review practices of outpatient ancillary staff such as medical assistants and technicians, though previous work in scribe documentation has shown that scribe chart review varies in completeness and data sources reviewed.^30^^,^^32^^,^^42^ Other ancillary staff operating in a team-based model may perform chart review with similar variability. While team-based workflows involving physicians and ancillary staff have been shown to increase efficient care in specialties such as cardiology and primary care,^26^^,^^27^^,^^43^ more studies are needed to determine the impact of ancillary staff in chart review and to establish best practices and guidelines for staff data review. Team-based care also presents opportunities for EHR redesign to augment collaborative workflows, which are currently not well-supported by EHRs.^44^ As ancillary staff continue to operate in an expanded capacity, EHR designs that support team-based documentation and chart review will be needed.

This study has limitations. First, only follow-up office visits for the 3 most common diagnoses for each subspecialty were analyzed. Further studies are needed to determine patterns of note access for other types of office visits and less common diagnoses. Second, audit logs are limited in their ability to capture clinical data and behaviors inside and outside the EHR. Our study aimed to characterize note review patterns using audit log data, though we recognize audit log data may not fully capture other sources of patient data such as summary views and does not include accesses to external imaging systems. Furthermore, we do not have data about note review habits prior to the implementation of EHRs as a comparison. Future work may include analyzing user accesses of other structured data sources.

## CONCLUSION

This study suggests that ophthalmologists and staff perform minimal manual note review and that note review is a collaborative practice. While our data are specific to an academic practice, our results call into question the clinical utility of progress notes for ophthalmology, and warrants further study in other specialties. Current time pressures and EHR inefficiencies may be contributing to workarounds that minimize time spent reviewing notes. Additional collaborations between physicians, ancillary staff, informaticians, and policymakers will be required to improve clinical documentation practices using EHRs and improve user-centered EHR designs that result in more efficient clinical care.

## FUNDING

This work was supported by the National Institutes of Health, Bethesda, MD [R00LM12238, P30EY10572] and by unrestricted departmental funding from Research to Prevent Blindness (New York, NY). JSC is also supported by a Research to Prevent Blindness Medical Student Fellowship (New York, NY). W-CL and AR are supported by a National Library of Medicine training grant from the National Institutes of Health (Bethesda, MD), T15LM007088.

## AUTHOR CONTRIBUTIONS

JSC—Study design, analysis and interpretation of data, and drafting and revising the manuscript.

MRH—Study design, data acquisition, analysis and interpretation of data, and drafting and revising the manuscript.

IHG—Study design, data acquisition, and analysis and interpretation of data.

AR—Interpretation of data and revisions.

W-CL—Interpretation of data and revisions.

HD—Interpretation of data and revisions.

MFC—Study design, interpretation of data, revising the manuscript, and supervision.

## CONFLICT OF INTEREST STATEMENT

MFC was previously a consultant for Novartis (Basel, Switzerland) and an equity owner in InTeleretina, LLC (Honolulu, HI). All other authors have no conflicts to disclose.

## DATA AVAILABILITY STATEMENT

The data underlying this article cannot be shared publicly to protect the privacy of individuals that were included in this study. The data will be shared on reasonable request to the corresponding author.
